# Comparison of Hybribio-H13 and Hybrid Capture® 2 human papillomavirus tests for detection of CIN2+ and CIN3+

**DOI:** 10.7705/biomedica.7061

**Published:** 2024-05-31

**Authors:** María Cecilia Agudelo, Edmundo Torres-González, Samuel Agudelo, Arianis Tatiana Ramírez, Kelly Melisa Castañeda, Connor J. Kinslow, María Rodríguez-Herrera, Lisa Garland, Yi Xie, Carlos Alberto Orozco, Mark Stoler, Michael Dean, Gloria Inés Sánchez

**Affiliations:** 1 Grupo Infección y Cáncer, Facultad de Medicina, Universidad de Antioquia, Medellín, Colombia Universidad de Antioquia Grupo Infección y Cáncer Facultad de Medicina Universidad de Antioquia Medellín Colombia; 2 Laboratory of Translational Genomics, Division of Cancer Epidemiology and Genetics, National Cancer Institute, Gaithersburg, MD, USA National Cancer Institute Laboratory of Translational Genomics Division of Cancer Epidemiology and Genetics National Cancer Institute, Gaithersburg MD USA; 3 Department of Radiation Oncology, Vagelos College of Physicians and Surgeons, Columbia University Irving Medical Center, New York, NY, USA Columbia University Irving Medical Center Department of Radiation Oncology Vagelos College of Physicians and Surgeons Columbia University Irving Medical Center New York, NY USA; 4 Department of Pathology, University of Virginia Health System, Charlottesville, VA, USA University of Virginia Health System Department of Pathology University of Virginia Health System Charlottesville, VA USA

**Keywords:** Uterine cervical neoplasms, human papillomavirus viruses, human papillomavirus DNA tests, neoplasias del cuello uterino, virus del papiloma humano, pruebas de ADN del virus del papiloma humano

## Abstract

**Introduction.:**

Low-cost, accurate high-risk HPV tests are needed for cervical cancer screening in limited-resource settings.

**Objective.:**

To compare the performance of the low-cost Hybribio-H13 test with the Hybrid Capture® 2 to detect cervical intraepithelial neoplasia grade 2 or 3 (CIN2 and CIN3).

**Materials and methods.:**

Archived baseline samples tested by the Hybrid Capture® 2 from women of the ASCUS-COL trial, aged 20 to 69 years, with biopsy-colposcopy directed diagnosis of CIN2+ (n = 143), CIN3+ (n = 51), and < CIN2 (n = 632) were blindly tested by the Hybribio-H13 test.

**Results.:**

The relative sensitivity of the Hybribio-H13 test versus the Hybrid Capture® 2 for detecting CIN2+ was 0.89 (90% CI = 0,80-0,98; NIT = 0,66), and for CIN3+ was 0,92 (90% CI = 0,85-0,98; NIT = 0,35). Relative specificity was 1.19 (90% CI = 1.05-1.33; NIT < 0.00001). In the analysis restricted to women older than 30 years, the relative sensitivity of the Hybribio-H13 for CIN3+ was marginally below unity (ratio = 0.97; 90% CI = 0.95-0.99), and the specificity remained higher than the Hybrid Capture® 2 test.

**Conclusion.:**

The Hybribio-H13 test was as specific as the Hybrid Capture® 2 for detecting CIN2+ or CIN3+ but less sensitive. Considering these results and the young age of the population recruited for screening because of ASCUS cytology, we suggest our results warrant the evaluation of the Hybribio-H13 for screening cervical cancer, especially in the evaluated population.

## Resumen

**Introducción.:**

Se necesitan pruebas para detectar genotipos de VPH de alto riesgo, precisas y de bajo costo, para la tamización del cáncer de cuello uterino en entornos de recursos limitados.

**Objetivo.:**

Comparar el desempeño de la prueba de bajo costo *Hybrid-H13* con la de *Hybrid Capture® 2* para detectar NIC2+ y NIC3+.

**Materiales y métodos.:**

Se analizaron en ciego muestras de la línea base provenientes de mujeres del estudio ASCUS-COL, entre los 20 y los 69 años, con diagnóstico dirigido por biopsia-colposcopia de NIC2+ (n = 143), NIC3 + (n = 51) y < NIC2 (n = 632) con la prueba para detección de virus de papiloma humano *Hybribio-H13.* Estas muestras fueron previamente evaluadas con la prueba *Hybrid Capture® 2*.

**Resultados.:**

La sensibilidad relativa de Hybribio-13 versus la de *Hybrid Capture® 2* para detectar NIC2+ fue de 0,89 (IC_90%_: 0,80-0,98; NIT = 0,66) y para NIC3+ fue de 0,92 (IC_90%_: 0,85-0,98; NIT = 0,35). La especificidad relativa fue de 1,19 (IC_90%_: 1,05-1,33; NIT < 0,00001). En el análisis restringido a mujeres mayores de 30 años, la sensibilidad relativa de Hybribio-H13 para NIC3+ estuvo marginalmente por debajo de la unidad (proporción = 0,97; IC_90%_: 0,95-0,99) y la especificidad permaneció más alta que la de la prueba *Hybrid Capture® 2*.

**Conclusión.:**

La prueba de *Hybribio-H13* fue tan específica como la de *Hybrid Capture® 2*, pero menos sensible para detectar NIC2+ o NIC3+. Teniendo en cuenta estos resultados y la temprana edad de la población reclutada en la tamización por la presencia de ASCUS en la citología, se sugiere continuar con la evaluación de la prueba *Hybribio-H13* para la detección de cáncer de cuello uterino en poblaciones con las mismas características que las de la aquí evaluada.

In 2020, there were 604,127 new cases and 341,831 deaths due to cervical cancer. Around 90% of these cases and deaths occur in Asia, Africa, Latin America, and the Caribbean regions ^1^. Human papillomavirus (HPV) is the cause of virtually all cervical cancers, with HPV types 16 and 18 accounting for approximately 70% of the cases ^2^. Prophylactic vaccination against HPV-16 and HPV-18 provides more than 90% protection against infection and associated high-grade lesions -like cervical intraepithelial neoplasia (CIN) grade 2 or 3 (CIN2 and CIN3)- or cancer ^3^. However, because currently implemented HPV vaccines do not eliminate the risk of cervical cancer, early detection remains a public health need.

Cytology-based screening is associated with an important reduction in the incidence and mortality of cervical cancer, especially in high-income countries but it has not achieved that impact in low-middle income countries**.** The main reason is the low sensitivity of cytology, which requires repeated testing that hinders required access to regular screening and follow-up to gynaecological management of positive results ^4^. Human papillomavirus testing has a sensitivity of around 100% to detect cervical high-grade lesions and has a high negative predictive value allowing the extension of screening to every five years. Other important attributes include automation, high reproducibility, and faster turnover of results than cytology ^5^. Therefore, HPV testing is an alternative currently available for cervical screening, especially in low-middle income countries where performer-dependable method implementation has been challenging. However, HPV testing has not been widely implemented in routine healthcare services of these countries. Most of the current HPV tests are expensive and require advanced equipment ^6^.

The Hybribio-13 HPV test from Hybribio (Hybribio Biotechnology Limited Corp., Hong Kong, China), hereafter referred to as H13, is a low-cost test based on a quantitative polymerase chain reaction (qPCR) that detects as a pool the HPV-16, 18, 31, 33, 35, 39, 45, 51, 52, 56, 58, 59, and 68 genotypes in cervical exfoliates ^7^. The H13 test does not require complex infrastructure and is robust, with an easy interpretation of results obtained in about three hours.

Two studies have compared the performance of the H13 test to detect CIN2+ with the reference standard HPV Hybrid Capture® 2 test (hereafter the HC2 test). In the study of 516 women with samples from the Kaiser Permanente Northern California (KPNC) repository, the agreement between H13 and HC2 was good since H13 correctly identified 91.5% of HPV-positive HC2 samples among CIN2+ cases and 92.1% of HPV-negative HC2 samples among < CIN2 ^7^. Within the framework of the Validation of HPV Genotyping Tests-3 (VALGENT-3) study -an established framework with a repository of 1,600 samples for evaluating HPV test clinical performance relative to validated comparators - researchers compared HC2 with a new version of H13 (then called H14), which, in addition to including the HPV 66 genotype, reports genotypes 16 and 18 individually. Relative sensitivity and specificity of H14 versus HC2 for detecting CIN2+ were 0.98 (95% CI = 0,94-1,03; NIT = 0,01) and 0,97 (95% CI = 0,96-0,99; NIT 0,78), respectively^8^.

Although these results suggest that H13 or H14 might be attractive for cervical cancer screening in low-resource settings given its low cost, no study has compared the performance of H13 or H14 to reference standards in samples of women from low-middle income countries. In this secondary analysis of the phase III randomized controlled ASC-US trial ^9^ we present a head-to- head comparison of the H13 assay with the reference QIAGEN© HC2 HPV DNA to the detect CIN2+ and CIN3+ in 842 women participating in this trial.

## Materials and methods

### Study design and population

**Supplementary figure 1.**
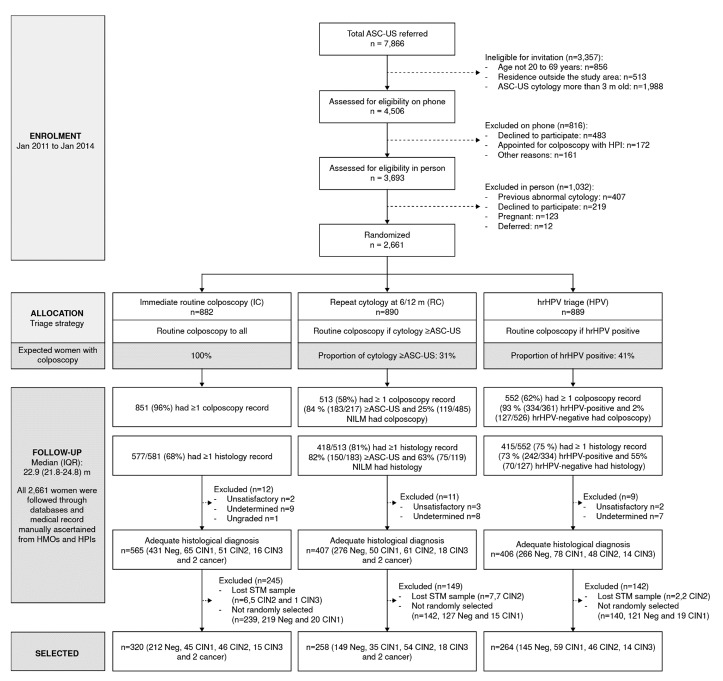
ASCUS-COL CONSORT flowchart indicates the number of women recruited and allocated in each arm. We account reports of cytology, colposcopy, and histology ascertained manually for all women from medical records or databases in the corresponding Health Maintenance Organization and History of Present Illness. The diagram shows the number of women with adequate histological diagnosis and retested by H13.

Samples for this study were selected from the ASCUS-COL trial. The ASC-US-COL is a three-arm, non-blinded, parallel-group pragmatic trial. Women aged 20 to 69 years (n = 2,661) with first-time presence of atypical squamous cells of undetermined significance (ASC-US) detected by cytology in the last two years were flagged in routine screening services and randomly allocated to receive immediate colposcopy (IC arm; n = 882), repeat cytology at 6 and 12 months (RC arm; n = 890), or an HPV test within two months of recruitment (HPV arm; n = 889). Colposcopy and biopsies, according to clinician judgment, were recommended for all women in the IC arm, for women with a repeat ASC-US or worse (ASC-US-positive) cytology in the RC arm, and a high-risk HPV test for women in the HPV arm. Hybrid Capture 2 HPV DNA test (HC2, Qiagen^™^, Germantown, USA) was conducted at the laboratory of infection and cancer at the *Universidad de Antioquia*. All women received invitations, and 80% (n = 2,132) attended the exit visit after 24 months of follow-up, which included high-risk HPV and cytology tests ^9^. All women positive for either test were referred to a certified, well-trained colposcopy specialist using a standardized and controlled protocol of biopsy sampling. After the end of the study, two blinded accredited experts confirmed the histopathological diagnoses of 1,407 women with at least one histological (n = 1.327) or endocervical (n = 80) diagnosis record, and the baseline samples of women in the IC and RC arms were tested for high-risk HPV by HC2 (Qiagen^™^) (supplementary figure 1). ASCUS-COL is registered with ClinicalTrials.gov (NCT02067468).

### Selection of participants for sub-study HC2 vs H13 comparison

Women identified after the end of the ASCUS-COL trial with biopsycolposcopy-directed, adequate diagnosis, and enough remaining archived baseline samples in Specimen Transport Medium^™^ (Qiagen^™^) for further testing (n = 1,348) were considered eligible for this study. We included all women diagnosed with CIN2+ (n = 197) and a representative sample of age- matched women (n = 645) with a final negative or CIN1 histological diagnosis, as shown in supplementary figure 1. The residual content of the specimen transport medium tube, used for the HC2 HPV DNA testing of the collected samples at the recruitment visit, was used for the H13 testing. Data collection and testing of the reference standard (histopathological diagnosis) and comparator test (HC2) were conducted before the index test (H13). The HC2 and H13 assays, as well as the verification of the histological diagnoses, were conducted independently and blindly.

**Figure 1.**
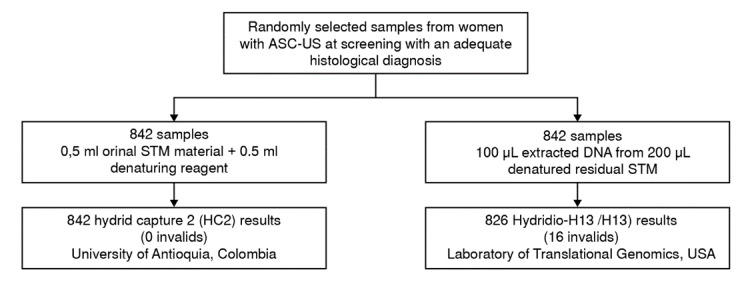
Flowchart showing the process from the sample panel collation and HPV testing to the final endpoint ascertainment of diseased and non-disease groups. STM: Specimen transport medium.

### Qiagen™ HC2 HPV DNA test

This test is based on a DNA-RNA hybridization that identifies a pool of 13 high-risk HPV genotypes (16, 18, 31, 33, 35, 39, 45, 51, 52, 56, 58, 59, and 68). Cervical cells were collected from women with a cytobrush (HC cervical sampler) and transferred to a tube containing 1 ml of Specimen Transport Medium^™^. The HC2 HPV DNA testing (Qiagen^™^, Gaithersburg, MD, USA) was performed according to the manufacturer’s instructions at the HPV laboratory of the *Grupo de Infección y C*á*ncer* at the *Universidad de Antioquia* (Medellín, Colombia). Relative light unit values -higher than one- were considered positive.

### HybriBio (H13) DNA extraction and testing

The DNA for the HybriBio-13 test was extracted from the denatured residual content of the specimen transport medium tube used for the HC2 HPV DNA test by a standard protocol ^10^. Briefly, each specimen was digested for two hours at 55 °C with 200 μg of proteinase K per ml and 1% Laureth-12. The samples were heated to 95 °C for 10 minutes to denature the residual protease. After precipitation with 5M ammonium acetate and 70% ethanol, DNA was washed, dried, and resuspended in 100 μl of TE buffer (10 mM TRIS + 0.1 mM EDTA) and frozen at -30 °C until shipped at room temperature to the Laboratory of Translational Genomics of the National Cancer Institute (NCI/ NIH, Bethesda, MD, USA), where testing was performed.

The H13 test is a real-time qPCR assay that uses specific primers and probes targeting the HPV E6 and the human β-globin genes. One of the probes is labeled with 6-carboxyfluorescein (FAM) fluorescent dye for the detection of a pool of 13 high-risk HPV genotypes and the other with (6-carboxy-4',5'-dichloro- 2',7'-dimethoxyfluorescein, HEX) fluorescent dye and shows human β-globin gene working as an internal control. The reagents’ volume and input DNA were modified from those described in the manufacturer’s instructions. The final volume was 11 μl, including 8.75 μl of PCR Master Mix kit, 0.25 μl of DNA Taq Polymerase, and 2 μl of the sample DNA. We have previously shown that this modification results in the minimum assay volume required with equivalent results ^11^. We used the positive and negative controls included in the H13 kit. A Ct value lower than or equal to 40 was the threshold considered as a positive result. Negative samples with no positive signal in the internal control were excluded from the analysis. The real-time instrument used was a Roche LightCycler 480 II.

### Sequencing of BSGP5+/6+ amplimers

To determine the HPV type of discordant samples (positive HC2/negative H13, n = 97, and negative HC2/positive H13, n = 29), DNA purified from exfoliates as described above was amplified with BSGP5+/6+ primers and the amplicon was sequenced with Sanger. The conserved BSGP5+/6+ primer pair amplifies a region of 150 bp of the L1 gene containing unique sequences that distinguish HPV genotypes ^12^^,^^13^. The sequences were used as a query for screening the GenBank database (www.ncbi.nlm.nih.gov) with BLAST Software 1. HPV types were assigned when we found a match between the 150 bp interprimer region and an HPV sequence in the GenBank.

### Sample size

We excluded 16 invalid samples -six negatives, seven CIN1, and three CIN2- because of unusual signals in the cellular internal control of the H13 test. The final analysis included 826 women. Based on the recommendations of Meijer *et al*. that at least 60 samples should be analyzed to assess whether a candidate test has a sensitivity for CIN2+ not less than 90% of that of HC2 ^14^, our study included 194 samples with CIN2+ for a power of 99.6% and 51 samples with CIN3+ for a power of 92.7%. Non-inferiority test (NTI) of H13 to HC2 concerning clinical specificity for < CIN2 was assessed in 632 cervical samples of women who did not have histologically confirmed CIN2+ with a power of 90%.

### Statistical methods

Sensitivity and specificity with corresponding 95% confidence intervals were estimated to detect CIN2+ or CIN3+ using < CIN2 (without neoplasia or CIN1) as disease-free categories. The McNemar test (McN) was applied to compare the differences between matched proportions. A matched noninferiority test (NIT) with a 90% relative sensitivity threshold and a 98% relative specificity threshold was applied to compare the clinical performance of the H13 to the HC2. Statistical significance for both statistics (McN and NIT) was set at 0.05. All analyses were conducted using the Stata 13 software (StataCorp LLC, Texas, USA).

### Ethical approval

ASCUS-COL complied with Colombian Resolution 8430 of 1993 to conduct studies in humans and followed the CIOMS guidelines ^15^. The ethics committees for human experimentation of the *Sede de Investigación Universitaria* (SIU) (Resolution 08-036-171) and the *Escuela de Medicina* (Resolution 004/2008) from the *Universidad de Antioquia* approved this study. Participants signed written informed consent, including authorization to use their samples and data for future research.

## Results

Specimens from 842 women, collected and previously tested with the HC2 at the enrolment visit, were selected from the 2,661 participants of the ASCUS-COL study with adequate histological diagnosis and retested by the H13 (figure 1). Sixteen (1.9%) samples of these residual specimens tested invalid with H13 and were excluded from further analysis, resulting in 826 samples analyzed by both the HC2 and H13 tests. The clinical features of the studied population are summarized in table 1. All women had an ASCUS pap smear for the first time at the screening visit. Most women were under 40 years old (75%), around half started regular sex between 16 and 19 years old, and 54% had between three and four lifetime sexual partners. Slightly more than half of the women included in this analysis had a definitive histological diagnosis at a six-month follow-up (57%).

**Table 1.** Demographic and clinical characteristics of the 842 women from the ASCUS-COL trial at the recruitment visit

Characteristic	n (%)
Age (years)	
20-29	379 (45.0)
30-39	252 (29.9)
40-49	151 (17.9)
≥ 50	60 (7.1)
Time to histological diagnosis (months)	
1-6	476 (56.5)
7-12	65 (7.7)
13-18	39 (4.6)
> 18	262 (31.1)
Age of first sexual intercourse (years)	
≤ 15	248 (29.4)
16-19	445 (52.9)
≥ 20	149 (17.7)
Number of lifetime sexual partners	
1-3	458 (54.4)
4-5	203 (24.1)
≥ 6	181 (21.5)
Histological diagnosis	
Negative	506 (60.1)
CIN1	139 (16.5)
CIN2	146 (17.3)
CIN3	47 (5.6)
SCC/ADC	4 (0.5)

CIN1: cervical intraepithelial neoplasia grade 1; CIN2: cervical intraepithelial neoplasia grade 2; CIN3: cervical intraepithelial neoplasia grade 3; SCC: squamous cell carcinoma; ADC: adenocarcinoma

The number of histological diagnoses in the 842 included participants were: 506 women without cervical lesions, 139 with CIN1, 146 with CIN2, 47 with CIN3, and four with cancer. HC2 and H13 tests were positive in 389 (60%) and 335 (52%) of the 645 women with < CIN2; and in 182 (92%) and 160 (81%) of the 197 CIN2+ cases, respectively. For the CIN3+ threshold, HC2 and H13 tests were positive in 48 (94%) and 44 (86%) of 51 women with CIN3+, respectively (supplementary table 1).

Sensitivity and specificity to detect CIN2+ or CIN3+ are shown in table 2. The H13 test showed a slightly higher specificity for < CIN2 (47% versus 39%; difference = 7.6; 95% CI = 4.6-10.6), and the HC2 test exhibited higher sensitivity to detect CIN2+ (93% versus 82%; difference = 10.3; 95% CI = 5.215.5) or CIN3+ (94% versus 86%; difference = 7.9; 95% CI = 0.5-15.2).

**Table 2.** Specificity and sensitivity of HC2 and H13 HPV tests for the detection of CIN2+ and CIN3+

			< CIN2 (n = 632)				CIN2+ (n = 194)				CIN3+ (n = 51)		
HPV tests		TN	FP	Specificity, % (95% CI)		TP	FN	Sensitivity, % (95% CI)		TP	FN	Sensitivity, % (95% CI)	
HC2		249	383	39.46	(35.62-43.40)	180	14	92.78	(88.18-96.01)	48	3	94.12	(83.76-98.77)
H13		297	335	47.00	(43.04-51.00)	160	34	82.47	(79.40-87.54)	44	7	86.27	(73.74-94.29)

CIN2: cervical intraepithelial neoplasia grade 2; CIN3: cervical intraepithelial neoplasia grade 3; HC2: Hybrid Capture^™^ 2 (Qiagen^™^); H13: Hybribio-H13 (Hybribio Biotechnology Limited Corp)

CI: Confidence interval; TN: true negative; FP: false positive; TP: true positive; FN: false negative

We excluded 16 samples (six negative biopsies, seven for CIN1, and three for CIN2) from analysis due to invalid results for H13 test.

Relative sensitivities for CIN2+ and CIN3+ and the relative specificity for < CIN2 of the H13 test compared to the HC2 test, were determined (table 3). In the analysis with all women (n = 826), H13 exhibits inferiority to HC2 with a relative sensitivity of at least 90% for CIN2+ (NIT = 0.6584) and CIN3+ (NIT = 0.3501). The H13 was non-inferior to HC2 with a relative specificity for < CIN2+ of at least 98% (NIT ≤ 0.00001). The relative sensitivity of H13 for CIN2+ and CIN3+ was below unity (ratio = 0.89; 90% CI = 0.80-0.98 and 0.92; 90% CI = 0.85-0.98), and the relative specificity for < CIN2 was significantly different from unity (ratio = 1.19; 90% CI 1.05-1.33). Similar results were found when restricting the analysis to women aged 30 and older (n = 454), the relative sensitivity of the H13 for CIN2+ and CIN3+ was below unity (ratio = 0.90; 90% CI = 0,81-0,98 and 0.92; 90% CI = 0,85-0,98), and the relative specificity for < CIN2 was (ratio = 1.11; 90% CI =0,99-1,24).

**Table 3.** Relative sensitivity for CIN2+ and CIN3+, and relative specificity for < CIN2 and CIN3+ for the H13 and the HC2 test comparison

		Relative sensitivity (90% CI)	Relative specificity (90% CI)	McN^a^	NIT^b^
All (n = 826)					
	CIN2+ (n = 194)	0,89 (0,80 - 0,98)		0,0002	0,6584
	CIN3+ (n = 51)	0,92 (0,85 - 0,98)		0,125	0,3501
	< CIN2 (n = 632)		1,19 (1,05 - 1,33)	<0,0001	<0,00001
≥ 30 years (n = 454)					
	CIN2+ (n = 106)	0,90 (0,81 - 0,98)		0,0212	0,5211
	CIN3+ (n = 31)	0,92 (0,85 - 0,98)		0,5000	0,2887
	< CIN2 (n = 348)		1,11 (0,99 - 1,24)	0,0039	0,0066

^a^ p for the McNemar test to set differences between matched proportions.

^b^ p for the non-inferiority test. A matched non-inferior statistic (ni) with a 90% relative sensitivity threshold and 98% relative specificity threshold was used to compare clinical performance of Hybribio-H13 (H-13) to Hybrid Capture^™^ 2 (HC2) tests.

Sixteen (six negative biopsies, seven CIN1, and three CIN2) samples, tested invalid by H13 test, were excluded from the analysis.

We further analyzed the discordance between the H13 and the HC2 test results by DNA sequencing (supplementary table 2). Among the 63 samples with high-risk HPV genotypes identified by sequencing, 50 (79.4%) were HC2+/H13-, and 13 (20.6%) were HC2-/H13+. Among the 23 samples that were negative or with low-risk HPV genotypes identified by sequencing, 16 (70%) were HC2+/H13- and 7 (30%) were HC2-/H13+.

## Discussion

In this study, we compared the clinical accuracy of the Hybribio-H13 test to the Hybrid Capture^™^ 2 (Qiagen^™^). Due to the matched design with samples tested with both H13 and HC2, we could calculate non-inferiority statistics. Samples were tested immediately after collection by the HC2 in Colombia and shipped to the USA, where testing by H13 was conducted using the minimum assay volume. Under these conditions, the H13 test did not conform to the acceptable standards of clinical performance for sensitivity to detect CIN2+ or CIN3+ but complied with the acceptable standards for specificity to detect < CIN2, overall, and in women of 30 years or older.

Currently, few studies properly comparing the clinical performance of H13 with standard reference tests have been published in peer-reviewed literature. A recent study described the clinical performance between the H13 and the HC2 tests in 373 samples from North America. The H13 correctly identified 94% of the HC2 HPV-positive CIN2+ cases and 88% of the HC2 HPV-negative cases ^7^. Likewise, in our study, The H13 identified 156 of the 180 (87%) HC2 CIN2+-positive cases and 224 of the 249 (90%) HC2 HPV-negative < CIN2 cases. In contrast to that description reporting 143/516 (28%) equivocal results, in our hands, the H13 test was highly robust, as the proportion of samples with equivocal results was very low (16/842; 1.9%).

In our study, HC2 samples were processed immediately after collection, and shortly after, manually extracted DNA was shipped at room temperature to the USA for the H13 testing. We cannot exclude the possibility that the differences between tests could be because of the modifications to the manufacturer’s instructions. Therefore, our results must be interpreted within the scope of this limitation. This study is the first in the international literature that presented a head-to-head comparison of the H13 assay with the reference Hybrid Capture^™^ 2 (Qiagen^™^) for the detection of CIN2+ and CIN3+ in a group of samples that allowed the performance of robust statistical tests with adequate power.

In this study, we included women with first-time ASCUS cytology at routine screening visits to healthcare services, 75% between 20 and 39 years of age. Under these conditions, the H13 test did not conform to the acceptable standards of clinical performance for sensitivity to detect CIN2 or CIN3+ but conformed for the specificity to detect < CIN2. In the analysis restricted to women aged 30 years or more, the relative sensitivity of the H13 for CIN3+ was marginally below unity (ratio = 0.97; 90% CI = 0.95-0.99), and the specificity remained higher than the HC2.

In conclusion, this study is the first to compare head-to-head the performance of the H13 test with a reference test such as the Hybrid Capture™ 2 (Qiagen^™^). The H13 test was as specific but less sensitive than HC2 to detect CIN2+ or CIN3+. Considering these results and the young age of the population recruited for screening due to ASCUS in the cytology, we suggest the H13 test is useful for screening cervical cancer, especially in women over 30 years who are subjected to screening with HPV tests according to Colombian clinical practice guidelines, and that these data contribute to the use of the H13 test as a screening method.

## Archivos suplementarios

**Supplementary table:** 1. Neoplasia per age range, and HC2 and H13 test results according to histological diagnosis

	Negative n = 506	CIN1 n = 139	CIN2 (n = 146)	CIN3 (n = 47)	CIN2 (n = 146)
	n (%)	n (%)	n (%)	n (%)	n (%)
Age (years)					
20-29	210 (41.5)	79 (56.8)	70 (47.9)	20 (42.6) 0	(0.0)
30-39	154 (30.4	31 (22.3)	49 (33.6)	17 (36.2) 1	(25.0)
40-49	104 (20.6)	18 (12.9)	19 (3.0)	8 (17.0) 2	(50.0)
≥50	38 (7.5)	11 (7.9)	8 (5.5)	2 (4.3) 1	(25.0)
HPV-HC2					
Positive	277 (54.7)	112 (80.6)	134 (91.8)	44 (93.6) 4	(100.0)
Negative	229 (45.3)	27 (19.4)	12 (8.2)	3 (6.4) 0	(0.0)
HPV-H13					
Positive	240 (47.4)	95 (68.3)	116 (79.5)	40 (85.1)	4 (100)
Negative	260 (51.4)	37 (26.6)	27 (18.5)	7 (14.9) 0	(0.0)
Equivocal	6 (1.2)	7 (5.0)	3 (2.1)	0 (0.0) 0	(0.0)

CIN1: cervical intraepithelial neoplasia grade 1; CIN2: cervical intraepithelial neoplasia grade 2; CIN3: cervical intraepithelial neoplasia grade 3; SCC: squamous cell carcinoma; ADC: adenocarcinoma; HPV-HC2: Hybrid Capture^™^ 2 human papillomavirus test; HPV-13: Hybribio 13-H13 human papillomavirus test

**Supplementary table 2.** HPV genotypes identification by sequencing in discordant HC2/H13 samples

HPV genotype by sequencing	Positive HC2 / Negative H13 n = 97	Negative HC2 / Positive H13 n = 29	Total
16, 31, 33, 45	50	13	63
6, 26, 30, 32, 53, 67,	16	7	23
87, 90 or negative			
Not obtained sequence	31	9	40

Sequencing analysis included the identification of 13 high-risk HPV types contained in the Hybrid Capture® 2 test (HPV 16, 18, 31, 33, 35, 39, 45, 51, 52, 56, 58, 59, or 68) and some low-risk HPV types. Sixteen samples (six negative biopsies, seven CIN1, and three CIN2) tested invalid by H13 and were excluded from the analysis. Genotype 66 was not considered since it was not found in either of the two tests.
